# Fabrication of bulk superhydrophobic wood by grafting porous poly(divinylbenzene) to wood structure using isocyanatoethyl methacrylate†

**DOI:** 10.1039/d4ra00889h

**Published:** 2024-05-10

**Authors:** Xinyu Fang, Ruijia Liao, Kaiji Wang, Miao Zheng, Hongji Li, Rui Wang, Xiaorong Liu, Youming Dong, Kaili Wang, Jianzhang Li

**Affiliations:** a Co-Innovation Center of Efficient Processing and Utilization of Forest Resources, College of Materials Science and Engineering, Nanjing Forestry University No. 159 Longpan Road Nanjing 210037 China wangkaili212@163.com; b Tengzhou Tostar Power Electronic Engineering Co. Ltd Zaozhuang 277000 China; c MOE Key Laboratory of Wooden Material Science and Application, Beijing Forestry University No. 35 Tsinghua East Road Beijing 100083 China lijzh@bjfu.edu.cn

## Abstract

Superhydrophobic treatment of wood can effectively reduce the interaction between wood and moisture, avoiding deformation, cracking, mould, and other defects caused by water absorption, which can extend the service life of wood and broaden the application field. Currently, the poor abrasion resistance of superhydrophobic wood is a crucial problem limiting its widespread application, and the preparation of superhydrophobic wood with robustness, abrasion resistance, and chemical resistance remains a huge challenge. In this work, robust bulk superhydrophobic wood with excellent abrasion resistance and chemical durability was fabricated by synthesizing porous poly(divinylbenzene) in wood cell cavities using graft copolymerization and solvothermal methods. The contact angles and rolling angles on the superhydrophobic wood surface were approximately 156° and 3°, respectively. Superhydrophobicity was carried through the entire structure of the wood. Even after severe damage by abrasion and sawing, as well as tests with organic solvents and harsh environments, the superhydrophobic properties of wood remained stable. Meanwhile, the superhydrophobic wood exhibited great self-cleaning and antifouling properties. In addition, the water uptake and dimensional stability of the wood were significantly improved. This work developed a simple, efficient, and durable strategy for the fabrication of superhydrophobic wood with robustness, abrasion resistance, and chemical resistance, which was expected to be applied to the wood industry to achieve the high-value applications of wood products and extend their service life.

## Abstract 2

Robust bulk superhydrophobic wood with super abrasion resistance was prepared using graft copolymerization and solvothermal methods.

## Graphical abstract

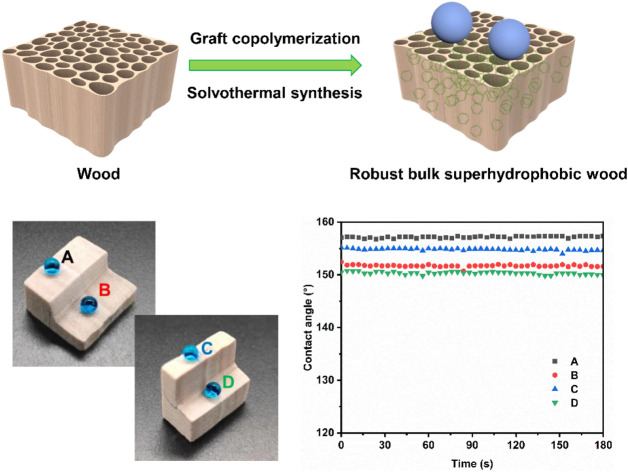

## Introduction

Of the four major raw materials recognized worldwide (wood, steel, cement, and plastics), wood is the only renewable resource. Due to its unique multi-scale hierarchical pore structure and chemical composition, wood possesses properties that are unparalleled by other materials, such as light weight and high strength, excellent processability, anisotropy, good thermal insulation, ultraviolet absorption and infrared reflection, as well as great environmental and acoustic properties, *etc.*, and is therefore widely used in home decoration, building structures, transportation, and other fields.^1–5^ Wood is a porous material mainly composed of cellulose, hemicellulose, lignin, *etc.*, with the presence of a large number of hydrophilic groups such as hydroxyl groups, and has extremely hydrophilic properties.^6,7^ The absorption of water by wood easily leads to size deformation, decay, discoloration, and even degradation, which seriously hinders its practical application.^8–10^

Superhydrophobic phenomena are widely found in nature, such as the “lotus leaf effect”, “rose petals”, “insect wings”, “water strider leg”, *etc.* Superhydrophobic surfaces, on which the contact angles (CAs) of a water droplet are greater than 150° and the rolling angles (RAs) are less than 10°, are closely scrutinized for their excellent water repellency, self-cleaning, antifouling, and anti-icing properties.^11–14^ Micro-/nano-rough structures and low surface energy substances act synergistically to determine the superhydrophobicity of material surfaces.^15,16^ Based on this, superhydrophobic surfaces can be constructed in two ways: one is to construct rough structures on the surface of low-energy materials, and the other is to modify low-energy substances on the micro-/nano-rough surfaces. The low-energy materials are usually fluorine-containing compounds, long-chain silane compounds, and so on.^17,18^ When preparing superhydrophobic surfaces, reducing the surface free energy of the material is easier to realize at the technical level, so the key is to construct suitable micro-/nano-rough structures. By bionically constructing a micro-/nano-rough structure on the wood substrate and subsequently treating it with low surface energy, the wood is transformed from hydrophilic to superhydrophobic, which can significantly attenuate the interaction between wood and water. The wood is always kept dry, which can effectively avoid a series of defects such as deformation, cracking, decay, mould, discoloration, degradation, *etc.* This has important research value and practical significance for the high value-added utilization and functionalization expansion of wood.

Mechanical stability and chemical durability are crucial problems affecting the practical application of superhydrophobic wood.^19,20^ Mechanical stability includes the interfacial bonding between the superhydrophobic coating and the wood substrate, and the strength of the micro-/nano-scale rough structure on the coating surface. The weak interfacial bonding between the coating and the wood substrate will lead to easy peeling off of the coating, and the poor strength of the micro-/nano-scale rough structure on the coating surface will lead to the micro-/nano-scale fine structure being damaged by external forces such as abrasion and scratches, which affects superhydrophobic performance.^21–24^ In addition, factors such as light, oil, acid, alkali, and organic solvent corrosion, high temperature and humidity, and dust will also affect the superhydrophobic performance.^25–28^

Many methods are currently being used to construct superhydrophobic wood, including the sol–gel method, hydrothermal method, wet-chemical method, chemical vapor deposition, coating method, layer-by-layer assembly, plasma, graft-copolymer, *etc.*^29–36^ Jia *et al.*^37^ fabricated a high-wear-resistance superhydrophobic wood by a facile, alkali-driven method using SiO_2_ nanoparticles and vinyltriethoxysilane. The superhydrophobic wood had CAs of 156.6° and RAs of 1.8°, and exhibited a high tolerance to harsh mechanical damages such as sandpaper abrasion. The CAs of the obtained wood dropped below 150° after 270 cm of sandpaper abrasion. Lu *et al.*^38^ prepared superhydrophobic wood surfaces *via in situ* synthesis of Cu_2_(OH)_3_Cl nano-flowers and impregnating phenol formaldehyde resin and stearic acid to improve chemical and mechanical durability. The CAs and RAs of the modified wood surface were approximately 163° and 5°, respectively. The wood samples lost superhydrophobicity at 8 abrasion cycles (20 cm per cycle), and after 20 repeated abrasion cycles, the CAs decreased to about 140°. Wang *et al.*^39^ prepared a superhydrophobic wood by grafting poly(2-(perfluorooctyl)ethyl methacrylate) onto wood by atom transfer radical polymerization. The resultant superhydrophobic wood exhibited excellent water resistance with CAs of 156° and hysteresis of 4°, and also showed abrasion resistance. However, the samples lost their superhydrophobicity after only 50 cycles of sandpaper abrasion testing (10 cm per cycle). Although the superhydrophobic wood reported in the above studies has good abrasion resistance, it is still difficult to meet the requirements for practical use. Therefore, there are still significant challenges to constructing superhydrophobic wood with robustness, wear resistance, and durability.

Herein, the robust bulk superhydrophobic wood was fabricated by synthesizing porous poly(divinylbenzene) (PDVB) in wood cell cavities using graft copolymerization and solvothermal methods. Utilizing 2-isocyanatoethyl methacrylate (IEMA) with bifunctional groups, its isocyanate groups can react with the hydroxyl groups of wood cell walls, and the carbon–carbon double bonds were copolymerized with porous PDVB. Thus, the porous PDVB filled with wood cell cavities can form covalent connections with the wood cell walls. As a polymerization monomer, divinylbenzene (DVB) was *in situ* polymerized in wood structure by vacuum impregnation and solvothermal method to form porous PDVB, which provided the necessary micro–nano roughness and low energy components for superhydrophobic properties. Meanwhile, the superhydrophobic PDVB could play a role in blocking moisture, further improving the water resistance and dimensional stability of wood. The graft copolymerization and solvothermal strategies have the following advantages: (1) the modified wood has an overall superhydrophobicity, and even if it was abraded, the exposed new surface remained superhydrophobic. (2) The graft copolymerization consumed a large number of hydroxyl groups of the wood, resulting in improved dimensional stability of the wood; (3) the interfacial compatibility between wood and porous PDVB was good, and the overall properties of superhydrophobic wood were more stable; (4) synthesis of porous PDVB in the pores of wood using solvothermal method to achieve superhydrophobicity, avoiding the use of expensive fluorine-containing or long-chain hydrophobic reagents, which are non-toxic, non-hazardous and inexpensive. This work overcomes the problem of poor abrasion resistance of the superhydrophobic layer of wood, which is the novelty of this study. The microstructure, chemical composition, hydrophobicity, mechanical stability, chemical durability, water uptake, and dimensional stability of resultant superhydrophobic wood were characterized and analyzed in depth.

## Experimental

### Materials

Poplar wood (*Populus* spp.) was cut into blocks with the dimensions of 10 mm (longitudinal) × 20 mm (tangential) × 20 mm (radial). All wood blocks were ultrasonically cleaned with an ethanol/water mixture, and dried in an oven at 100 °C for 24 h until a constant weight was reached. 2-Isocyanatoethyl methacrylate (IEMA, 98%), divinylbenzene (DVB, 80%), 2,2′-azobis(2-methyl propionitrile) (AIBN, 98%), and dibutyltin dilaurate (DBTL, 95%) were purchased from Macklin Biochemical Co., Ltd (Shanghai, China). Ethyl acetate (99%), dimethylsulfoxide (DMSO, 99.8%), hydrochloric acid (HCl, 36–38%), sodium hydroxide (NaOH, 96%), sodium chloride (NaCl, 99.5%), toluene (99.5%), acetone (99.5%), *n*,*n*-dimethylformamide (DMF, 99.5%), decane (98%), 1,4-dioxane (99%), absolute ethanol were purchased from Sinopharm Chemical Reagent Co., Ltd (Shanghai, China).

### Preparation of IEMA wood

25 g of IEMA was dissolved in 500 mL of DMSO, 0.25 g of DBTL was added as a catalyst, and stirred vigorously to make a homogeneous mixture. The wood blocks were submerged in the above solution and vacuum impregnated for 30 min, and then placed in the atmosphere for 2 h. The whole system of wood and solution was placed in a three-necked flask, passed through nitrogen for 10 min, and then the reaction was continuously stirred at 85 °C for 4 h. The wood blocks were cleaned with DMSO and dried in an oven at 60 °C. The prepared wood samples were labeled as IEMA wood.

### Preparation of superhydrophobic wood

8 g of DVB was dissolved in 70 mL of ethyl acetate, 0.4 g of AIBN was added, and ultrasonicated in an ice bath for 30 min. The IEMA wood and original wood blocks were immersed in the solution, respectively, and vacuum-impregnated for 30 min, then placed in the atmosphere for 2 h. The whole system was transferred to a hydrothermal reactor and reacted at 100 °C for 24 h. The wood blocks were washed with ethyl acetate for 3 times and dried in an oven at 45 °C to obtain the superhydrophobic wood. Superhydrophobic wood prepared by hydrothermal treatment using original wood is labeled as PDVB wood and using IEMA wood is labeled as IEMA–PDVB wood.

### Characterization

The microstructures of wood samples were characterized using a scanning electron microscope (SEM, SU8010, Hitachi). The chemical components of wood samples were performed by a Fourier transform infrared (FTIR, Nicolet iS50) spectrometer with attenuated total reflection (ATR) mode. The water contact angles (CAs) and rolling angles (RAs) on wood samples were measured with a contact angle system, OCA20 (Dataphysics, Germany). The thermal degradation behaviors of the wood samples were conducted using a TGA55 thermogravimetric analyzer at a constant heating rate of 10 °C min^−1^ from 30 to 600 °C under a nitrogen atmosphere.

Abrasion resistance test: the abrasion resistance was characterized using tests of sandpaper wearing, sawing, and water jet washing. For the sandpaper-wearing test, the superhydrophobic wood was abraded with sandpaper (100 mesh) under the weight of 500 g. The abrasion was performed 100 times with an abrading distance of approximately 20 cm. For the sawing test, a hacksaw blade was used to repeatedly saw the superhydrophobic wood to expose a new surface. For the water jet washing test, the superhydrophobic wood was placed under a faucet and washed for 10 min. The variations of CAs after mechanical abrasion were measured.

Chemical and environment stability tests: the superhydrophobic wood samples were respectively immersed in a 1 M HCl solution, 1 M NaOH solution, saturated NaCl solution, toluene, acetone, ethanol, ethyl acetate, DMF, *n*-decane, and dioxane for 24 h. The superhydrophobic wood samples were placed at a high temperature of 100 °C and a low temperature of −30 °C for 24 h. The superhydrophobic wood samples were immersed in deionized water for 2 h under ultrasonication (frequency of 40 kHz, 100 W). The superhydrophobic wood samples were irradiated under a UV lamp (approximately 10 mW cm^−2^) with a wavenumber of 340 nm for 24 h at a distance of 10 cm. All the wood samples were dried at 100 °C for 3 h for subsequent hydrophobicity testing.

Water uptake and dimensional stability: all the wood samples were immersed in water at room temperature, and the weights and dimensions were measured after different time intervals (4, 12, 24, 48, 72, 96, 120, and 144 h). WU was calculated according to the formula:

1 WU (%) = (*W*_2_ − *W*_1_)/*W*_1_ × 100

where *W*_1_ denotes the weight of the oven-dried wood sample, and *W*_2_ denotes the sample weight after immersion in water.

The volumetric swelling (*S*) was calculated according to the formula:

2 *S* (%) = (*S*_2_ − *S*_1_)/*S*_1_ × 100

where *S*_1_ denotes the volume of the oven-dried wood sample, and *S*_2_ denotes the volume of the water-saturated wood sample.

Thereafter, the anti-swelling efficiency (ASE) was calculated based on the swelling difference between the original wood and treated samples (IEMA wood, PDVB wood, and IEMA–PDVB wood) as follows:

3 ASE (%) = (*S*_o_ − *S*_t_)/*S*_o_ × 100

where *S*_o_ and *S*_t_ are used for volumetric swelling of the original wood and treated wood samples, respectively.

## Results and discussion

### Preparation process and reaction mechanism

**Fig. 1:**
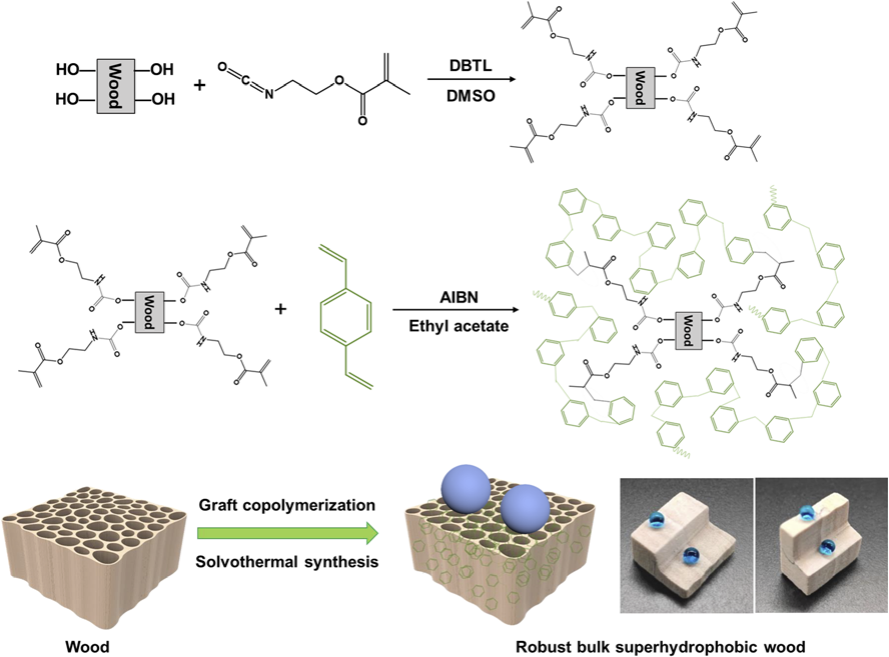
Preparation process and reaction mechanism of robust superhydrophobic wood.

The superhydrophobic wood is prepared by *in situ* polymerization of porous PDVB in wood cell cavities using graft copolymerization and solvothermal methods, as shown in Fig. 1. First, carbon–carbon double bonds were grafted to the wood cell walls by the reaction between the isocyanate groups of IEMA and the hydroxyl groups of the wood. Then, porous PDVB was synthesized in the wood cell cavities using a solvent-thermal method, and covalently bonded to the wood cell walls by copolymerization with the carbon–carbon double bonds, resulting in the robust bulk superhydrophobic wood.

### Superhydrophobic performance

**Fig. 2:**
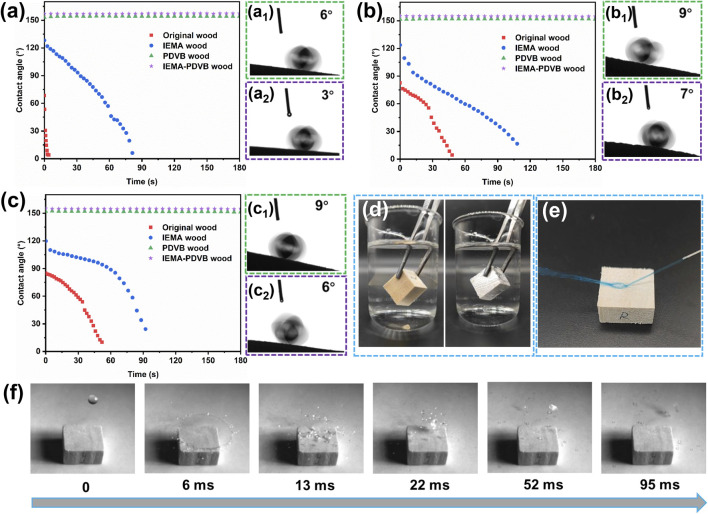
Superhydrophobic performance of wood samples: (a, b, c) contact angles as a function of time for original wood, IEMA wood, PDVB wood, and IEMA–PDVB wood in cross-section, radial section, and tangential section, respectively; (a_1_, a_2_, b_1_, b_2_, c_1_, c_2_) rolling angles for PDVB wood and IEMA–PDVB wood in cross-section, radial section, and tangential section, respectively; (d) mirror-like phenomenon, the left is original wood and the right is IEMA–PDVB wood; (e) jet water bounced on the surface of IEMA–PDVB wood; (f) photos of water droplet impacting the surface of IEMA–PDVB wood from a 100 cm height.

Fig. 2 illustrates the water wettability of different wood samples. The water droplets spread and penetrated rapidly on the cross-section of the original wood, and penetrated into the wood within 1 minute on the radial and tangential sections. Compared to the original wood, the hydrophobicity of the IEMA wood was improved by grafting double bonds through the chemical reaction with IEMA, with the initial CAs elevated to over 120° and the time required for penetration into the wood becoming longer. After the *in situ* polymerization of the porous PDVB in the pores of the wood, the porous PDVB provided the necessary micro–nano rough structure and low-energy components for wood superhydrophobicity, and the CAs of both PDVB wood and IEMA–PDVB wood were higher than 150°, and the RAs were less than 10°, achieving superhydrophobicity. Moreover, the superhydrophobicity of IEMA–PDVB wood was slightly better than that of PDVB wood. Compared to the original wood, the IEMA–PDVB wood exhibited a mirror-like phenomenon underwater when observed at an oblique angle (Fig. 2d), which is a typical characteristic of superhydrophobic surface. Superhydrophobic surfaces can trap air, and form a solid–liquid–air interface that effectively keeps underwater surfaces dry. When the water was dropped at a high-speed onto the IEMA–PDVB wood surface, the water droplet would bounce (Fig. 2e and Video S1†). As can also be seen from the high-speed camera images, water droplets falling from a height of 100 cm onto the surface of the IEMA–PDVB wood were completely bounced, leaving the surface dry. These results indicated the IEMA–PDVB wood has excellent repellence to water.

### Microstructure analysis

**Fig. 3:**
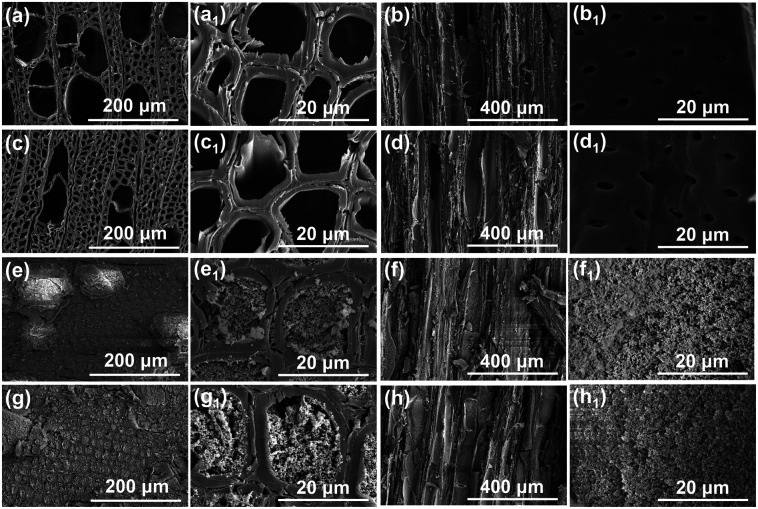
Micromorphology of wood samples in cross-section and longitudinal sections: (a, a_1_, c, c_1_, e, e_1_, g, g_1_) micromorphology of the cross-section of original wood, IEMA wood, PDVB wood, and IEMA–PDVB wood, respectively; (b, b_1_, d, d_1_, f, f_1_, h, h_1_) micromorphology of the longitudinal section of original wood, IEMA wood, PDVB wood, and IEMA–PDVB wood, respectively.

The micromorphology of original wood, IEMA wood, PDVB wood, and IEMA–PDVB wood in cross-section and longitudinal sections is shown in Fig. 3. Note that the wood surface for SEM characterization was cut flat by a microtome. The original wood showed a honeycomb cell structure in cross-section (Fig. 3a and a_1_), and in the longitudinal section, the vessel walls can be observed (Fig. 3b and b_1_). After IEMA treatment, the cell structure of IEMA wood appeared slightly crumpled and deformed (Fig. 3c, c_1_, d and d_1_). After polymerization of PDVB using a solvothermal method, the PDVB-wood and IEMA–PDVB wood exhibited similar micromorphology, that the porous PDVB filled into the wood cell cavities (Fig. 3c, c_1_, d, d_1_, g, g_1_, h and h_1_). The porous PDVB filled with wood cell cavities not only provided both a micro-/nano-rough structure, but reduced the surface energy of the modified wood, which is crucial to achieving superhydrophobicity.

**Fig. 4:**
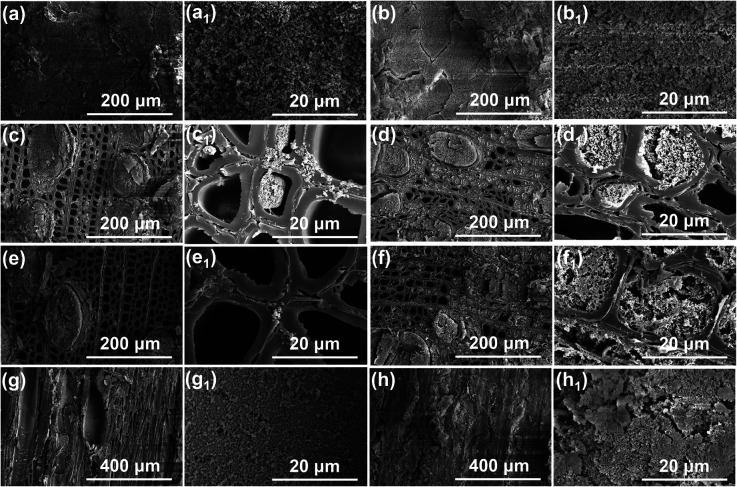
Micromorphology comparison of PDVB wood and IEMA–PDVB wood: (a, a_1_, b, b_1_) micromorphology of the cross-section of PDVB wood and IEMA–PDVB wood without cutting flat treatment by a microtome; (c, c_1_, d, d_1_ and e, e_1_, f, f_1_) micromorphology of the cross-section of PDVB wood and IEMA–PDVB wood with a thickness of 2.5 mm and 5.0 mm were removed by microtome, respectively; (g, g_1_, h, h_1_) micromorphology of the central layer longitudinal section of PDVB wood and IEMA–PDVB wood.

To reflect the difference caused by IEMA treatment or not on the micromorphology of PDVB wood and IEMA–PDVB wood, the cross-sections of the two samples were cut with different thicknesses, and the center layer of the longitudinal section was also cut to observe the micromorphology of the internal surface of these two wood samples. As shown in Fig. 4a, a_1_, b and b_1_, the outer surfaces of PDVB wood and IEMA–PDVB wood (the surface has not been smoothed by a microtome) showed a similar microstructure, with a large amount of porous PDVB covering the wood surface. However, the structural variability was exhibited when wood cross-sections were removed at different thicknesses. As shown in Fig. 4c, c_1_, d and d_1_, when a 2.5 mm thickness was removed from the wood cross-section, compared to the PDVB wood, more porous PDVB filled the cell cavities of the IEMA–PDVB, which was more pronounced with a 5.0 mm thickness removed. Moreover, the difference in the amount of PDVB filling was similarly reflected in the center layer of the longitudinal sections of the two samples, as shown in Fig. 4g, g_1_, h and h_1_, a larger amount of porous PDVB was deposited on the vessel walls of the IEMA–PDVB wood sample. This is probably because after the wood was chemically grafted by IEMA, more divinylbenzene monomers penetrated into the interior of the wood and could be covalently attached to the wood pore structure through a copolymerization reaction. Additionally, the BET surface area and porosity of the PDVB wood and IEMA–PDVB wood were provided, as shown in Fig. S1.† The BET surface area of the PDVB wood and IEMA–PDVB wood were 99.7923 m^2^ g^−1^ and 214.6327 m^2^ g^−1^, and their pore width distributions were similar. The BET results can also confirm that, compared to the PDVB wood, the porous PDVB was deposited in greater amounts inside the IEMA–PDVB wood. Therefore, based on microstructural analysis, IEMA–PDVB wood is expected to achieve robust bulk superhydrophobic properties.

### Chemical component analysis

**Fig. 5:**
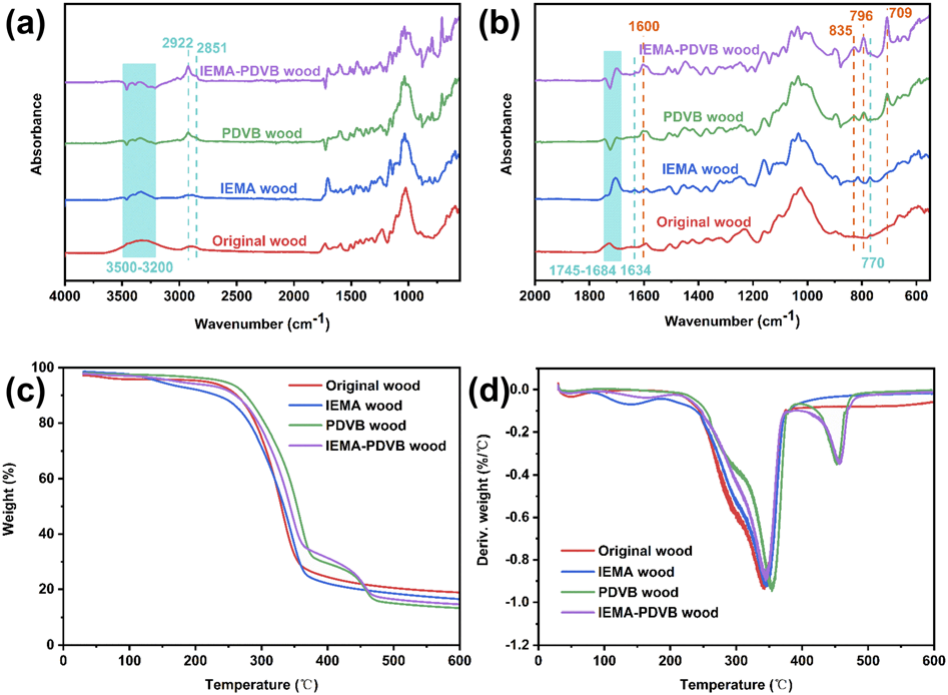
Chemical components: (a and b) FTIR spectra of original wood, IEMA wood, PDVB wood, and IEMA–PDVB wood; (c and d) TG and DTG curves of original wood, IEMA wood, PDVB wood, and IEMA–PDVB wood.

Fig. 5a and b display the FTIR spectra of original wood, IEMA wood, PDVB wood, and IEMA–PDVB wood. Compared to the FTIR spectrum of the original wood, the new peak characteristic peaks at 1634 cm^−1^ and 770 cm^−1^ appeared in IEMA wood, which were assigned the alkene stretching vibration and N–H bending vibration, respectively.^40^ Furthermore, the carbonyl peak at 1708 cm^−1^ was enhanced, and the band of the hydroxyl group (3500–3200 cm^−1^) was decreased.^41^ The above results indicated that the isocyanate groups of IEMA successfully reacted with the hydroxyl groups of the wood, thus grafting the carbon–carbon double groups to the wood. For the PDVB wood and IEMA–PDVB wood, the characteristic peaks at 709, 796, and 835 cm^−1^ are meta- and para-substituted aromatic rings and vinyl groups, respectively.^42,43^ The peak at 1600 cm^−1^ is the strong C=C stretching vibration of PDVB.^44,45^ These results demonstrated the successful polymerization of PDVB in wood. Moreover, the intensity of these characteristic peaks of PDVB in IEMA–PDVB wood was higher. Besides, the polymerization of PDVB in wood was further confirmed by TG/DTG curves, as shown in Fig. 5c and d. Compared with original wood and IEMA wood, TG/DTG curves of PDVB wood and IEMA–PDVB wood exhibited a new degradation region at 400–500 °C, which was caused by the degradation of PDVB.^46^

### Mechanical stability and chemical durability

**Fig. 6:**
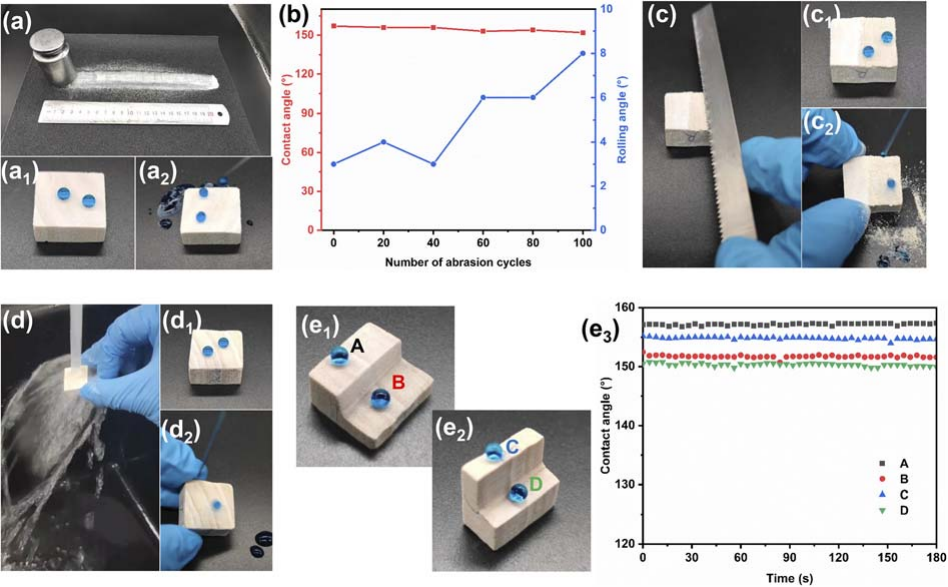
Mechanical stability of IEMA–PDVB wood: (a) sandpaper abrasion test; (a_1_, a_2_) static and roll-off diagrams of water droplets on abraded surfaces of sandpaper; (b) CAs as a function of number of abrasion cycles for IEMA–PDVB wood surfaces; (c) sawing test; (c_1_, c_2_) static and roll-off diagrams of water droplets on sawn surfaces; (d) water jet washing test; (d_1_, d_2_) static and roll-off diagrams of water droplets on water jet washing surfaces; (e_1_, e_2_) spherical water droplets resting stably on IEMA–PDVB wood surfaces and cut interior surface in cross-section and longitudinal section; (e_3_) contact angles as a function of time for the four positions of ABCD in (e_1_) and (e_2_).

The main limitation to the practical application of superhydrophobic wood is its poor abrasion resistance. Porous PDVB was synthesized in the pores of wood using graft copolymerization and solvothermal methods. The superhydrophobicity of the wood is present throughout the whole structure, resulting in excellent abrasion resistance properties. Fig. 6a and Video S2† show a sandpaper abrasion test of IEMA–PDVB wood, where the sample maintained excellent superhydrophobicity even after being subjected to abrasion over a total length of 20 meters. Fig. 6c and Video S3† exhibit the exposed new surface of the IEMA–PDVB wood by sawing with a saw blade that still has excellent water repellency. After placing the IEMA–PDVB wood under a faucet with water for 10 minutes, the surface was not wetted and remained water repellent ability (Fig. 6d and Video S4†). To verify the overall superhydrophobicity of the IEMA–PDVB wood, we performed transverse and longitudinal sawing along the center of the wood block and tested the exposed interior surfaces for dynamic CAs, the results are shown in Fig. 6e_1_–e_3_. The dynamic CAs of the exposed interior surfaces, both in cross-section and longitudinal section, remained stable for 180 s and were above 150°, indicating that the superhydrophobicity of IEMA–PDVB wood is indeed throughout the whole structure. The bulk superhydrophobic wood has excellent superhydrophobic properties throughout its service life, even after severe wear, which is difficult to achieve with conventional superhydrophobic wood.

The abrasion resistance of superhydrophobic wood in this study is compared with that reported in other literature, and the results are shown in Table S1.† The abrasion resistance of superhydrophobic wood constructed by the coating method is generally poor, and the superhydrophobicity is lost after sandpaper abrasion of about 1 meter length, whereas the superhydrophobic wood in this work can withstand sandpaper abrasion of 20 meters in length. Meanwhile, for bulk superhydrophobic wood, the superhydrophobic property throughout the whole wood structure, and has advantages in abrasion resistance. Superhydrophobicity cannot be achieved by direct chemical grafting of hydrophobic long chains, but bulk superhydrophobicity can be achieved by constructing roughness with nanoparticles and endowing low-energy components. In this work, porous PDVB was synthesized in the pore structure of wood to realize the bulk superhydrophobicity, which provided a new idea for the fabrication of bulk superhydrophobic wood.

**Fig. 7:**
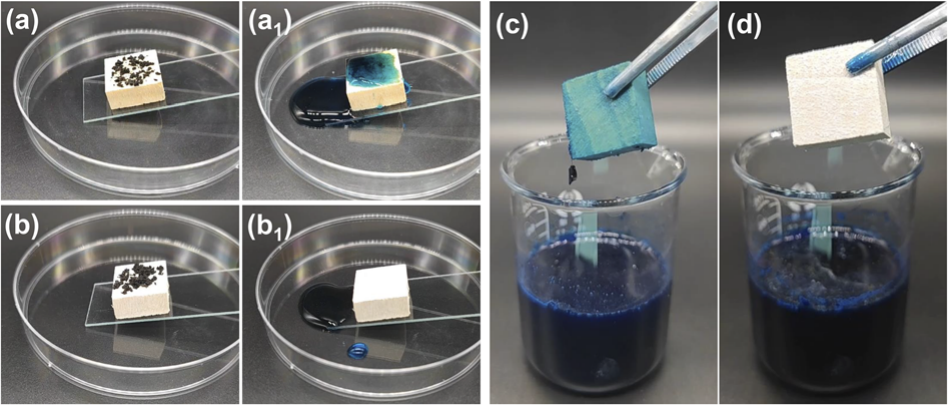
Self-cleaning and antifouling properties: (a, a_1_) images of the self-cleaning process on original wood surfaces; (b, b_1_) images of the self-cleaning process on IEMA–PDVB wood surfaces; (c, d) antifouling properties of the original wood and IEMA–PDVB wood.

The excellent water repellency of superhydrophobic material surfaces gives them great self-cleaning and antifouling properties. The self-cleaning behavior was performed by a dirt-removal test (Fig. 7a, a_1_, b and b_1_, and Video S5†), where the water is dropped onto a wood surface that is contaminated with carbon powder. For the original wood, water drops fell onto the surface and merged with the carbon powder, adhering to the wood surface and leaving a dirty and wet surface. For the IEMA–PDVB wood, the water droplets readily rolled off, removing the dirt and leaving a clean and dry surface. The original wood and IEMA–PDVB wood were dipped into dyed sewage, stirred and taken out, it could be observed that the original wood surface was stained with the sewage, whereas the IEMA–PDVB wood maintained a clean surface (Fig. 7c and d and Video S6†). The above tests indicated that IEMA–PDVB wood has excellent self-cleaning and anti-fouling properties.

**Fig. 8:**
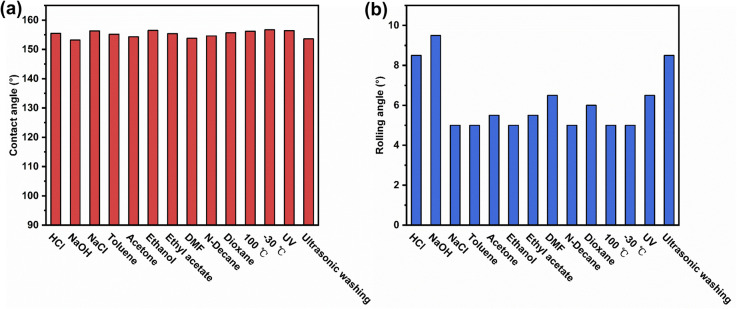
Chemical and environment durability of IEMA–PDVB wood: (a) CAs for IEMA–PDVB wood subjected to a variety of harsh chemical and environmental tests; (b) RAs for IEMA–PDVB wood subjected to a variety of harsh chemical and environmental tests.

In addition to mechanical stability, the chemical and environmental durability of superhydrophobic wood has important implications for its practical application. A variety of harsh chemical and environmental tests were performed to evaluate the durability of IEMA–PDVB wood. Fig. 8a and b show the CAs and RAs of the IEMA–PDVB wood after immersion in different chemical reagents (HCl solution of pH 2, NaOH solution of pH 12, NaCl saturated salt solution, toluene, acetone, ethanol, ethyl acetate, DMF, *n*-decane, and dioxane) for 24 hours, the CAs were all above 150° and the RAs were all below 10°. The IEMA–PDVB wood was placed at 100 °C and −30 °C for 24 hours and still maintained excellent superhydrophobicity. In addition, The IEMA–PDVB wood also remained stable superhydrophobicity after being subjected to 2 hours of ultrasonic cleaning and 24 hours of UV irradiation. These results demonstrated great chemical and environmental durability.

### WU and ASE

**Fig. 9:**
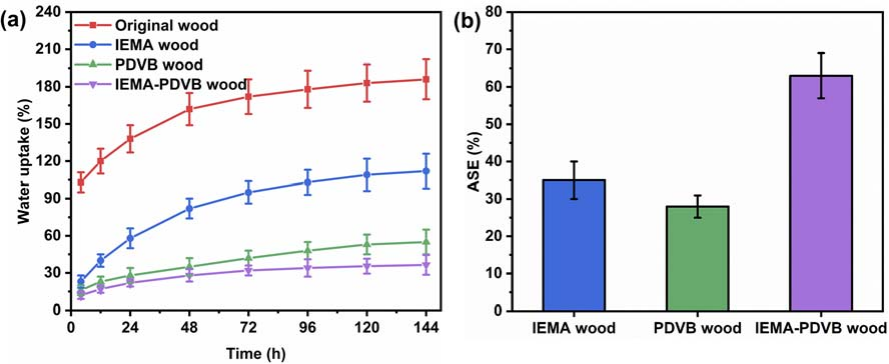
Water uptake capacity (a) and dimensional stability (b) of wood samples.

Fig. 9a shows the WU capacity of the original wood, IEMA wood, PDVB wood, and IEMA–PDVB wood. After 24 hours of immersion, the WU value of the original wood reached 138%, in contrast to the IEMA wood which showed 58% water absorption, the PDVB wood with 28% and IEMA–PDVB wood with 22% water absorption. After 144 hours of continuous immersion, the WU of the original wood, IEMA wood, PDVB wood, and IEMA–PDVB wood were 186%, 112%, 55%, and 36%, respectively. The decrease in WU of the IEMA wood was attributed to the reaction of the isocyanate groups of IEMA with the hydroxyl groups in the wood, which consumed a significant amount of the hydrophilic groups. The reduction in WU of superhydrophobic PDVB wood was due to its excellent water-repelling ability, which significantly reduced the amount of water that entered into the wood, thus avoiding the interaction between the wood and water. The lowest water absorption of IEMA–PDVB was because of the synergistic effect of hydroxyl consumption and water repellency. Fig. 9b shows the ASE values of IEMA wood, PDVB wood, and IEMA–PDVB wood for evaluating the dimensional stability of the samples. The ASE values of IEMA wood, PDVB wood, and IEMA–PDVB wood were 35%, 28%, and 63%, respectively, of which the IEMA–PDVB wood exhibited the best dimensional stability. For the IEMA–PDVB wood, a superhydrophobic PDVB was filled into the wood cell cavities while anchored to the cell walls by covalent bonding using graft copolymerization and solvothermal methods, not only consuming a large number of hydroxyl groups but endowing wood with water repellency, which was responsible for the significant increase in dimensional stability of IEMA–PDVB wood.

## Conclusions

In summary, the robust bulk superhydrophobic wood with excellent mechanical stability and chemical durability was fabricated by covalently anchoring porous PDVB to the wood cell walls using graft copolymerization and solvothermal strategies. The CAs and RAs were 156° and 3°, respectively. Due to its superhydrophobicity throughout the wood's whole structure, superhydrophobic wood could maintain excellent superhydrophobic properties even after being subjected to sandpaper abrasion (20 meters of length), sawing, and water jet washing. Meanwhile, the superhydrophobicity of wood was not affected by harsh chemical and environmental conditions tests such as immersion in chemical solvents, high and low temperatures, ultrasonic cleaning, and UV irradiation. In addition, the water absorption of the IEMA–PDVB wood was remarkably reduced and the dimensional stability was significantly improved. This work has addressed the problems of poor abrasion resistance and durability in the practical application of superhydrophobic wood, which is of great significance for its large-scale, high-value, and functionalized application.

## Author contributions

X. Fang: conceptualization, investigation, methodology, formal analysis, data curation, writing – original draft; R. Liao: methodology, formal analysis, data curation, writing – original draft; K. Wang: formal analysis, data curation; M. Zheng: methodology, formal analysis, data curation; H. Li: investigation, formal analysis, data curation; R. Wang: formal analysis, data curation; X. Liu: investigation, methodology, formal analysis, data curation; Y. Dong: investigation, methodology, formal analysis, data curation; K. Wang: conceptualization, supervision, project administration, funding acquisition, writing – review & editing; J. Li: supervision, project administration, writing – review & editing.

## Conflicts of interest

There are no conflicts to declare.

## Supplementary Material

RA-014-D4RA00889H-s001

RA-014-D4RA00889H-s002

RA-014-D4RA00889H-s003

RA-014-D4RA00889H-s004

RA-014-D4RA00889H-s005

RA-014-D4RA00889H-s006

RA-014-D4RA00889H-s007
