# Supplementary material for: Fabrication of bulk superhydrophobic wood by grafting porous poly(divinylbenzene) to wood structure using isocyanatoethyl methacrylate
Source: RSC Adv. 2024 May 10;14(22):15201–8. doi: 10.1039/d4ra00889h (PMC11082725; doi:10.1039/d4ra00889h)
Supplement: RA-014-D4RA00889H-s001 [file RA-014-D4RA00889H-s001.pdf]

Electronic Supplementary Material (ESI) for RSC Advances.
This journal is © The Royal Society of Chemistry 2024

Supporting Information

Fabrication of bulk superhydrophobic wood by grafting porous poly(divinylbenzene) to wood structure using isocyanatoethyl methacrylate

Xinyu Fang[a], Ruijia Liao[a], Kaiji Wang[b], Miao Zheng[a], Hongji Li[a], Rui Wang[a], Xiaorong Liu[a], Youming Dong[a], Kaili Wang[a*], Jianzhang Li[a, c*]

[a]Co-Innovation Center of Efficient Processing and Utilization of Forest Resources, College of Materials Science and Engineering, Nanjing Forestry University, No.159 Longpan Road, Nanjing 210037, China..

[b]Tengzhou Tostar Power Electronic Engineering Co. Ltd, Zaozhuang 277000, China.

[c]MOE Key Laboratory of Wooden Material Science and Application, Beijing Forestry University, No.35 Tsinghua East Road, Beijing 100083, China.

Table S1. The comparison of superhydrophobic wood in this work and previous studies.

Specimen	Method	CA (°)	RA (°)	Abrasion resistance	Bulk or coating	Ref.
Chinese fir	SiO_2 NPs modified by VTES	154	0	From 155° decreased to 151° with the sandpaper abrasion length of 1.2 m	Coating	1
Chinese fir	Alkali-driven SiO_2 NPs modified by VTES	157	2	From 157° decreased to 150° with the sandpaper abrasion length of 270 cm	Coating	2
Larch wood	Hydrothermal pre-treatment and deposit ZnO nanostructures	160	4	From 160° decreased to 140° with the sandpaper abrasion length of 50 cm	Coating	3
Masson pine wood	Hierarchical core/shell structures fabricated by Cu_2O NPs, PF resin, and stearic acid	153	-	From 153° decreased to 100° with the sandpaper abrasion length of 100 cm	Coating	4
Radiata pine wood	ZIF-8/paraffin with Hexadecyltrimethoxysilane	153	-	From 153° decreased to 150° with the sandpaper abrasion length of 100 cm	Coating	5
Pinus wood	Epoxy/$Cu_2(OH)_3Cl$ NPs/stearic acid	157	9	From 157° decreased to 130° with the sandpaper abrasion	Coating	6

				length of 400 cm		
Poplar wood	Liquid-vapor phase deposition of methyltrimethoxysilane	153	6	From 153° decreased to 150° with the sandpaper abrasion length of 100 cm	Coating	7
Radiata pine wood	Grafting long-chain stearoyl chloride	138	-	137° in internal surface	Bulk highly hydrophobic	8
Poplar wood	ZnO rods modified by palmitoyl chloride	155	3	151° in internal surface	Bulk superhydrophobic	9
Poplar wood	Porous PDVB	156	3	151° in internal surface	Bulk superhydrophobic	This work

1. Jia S, Liu M, Wu Y, et al. Facile and scalable preparation of highly wear-resistance superhydrophobic surface on wood substrates using silica nanoparticles modified by VTES. Applied Surface Science, 2016, 386: 115-124.
2. Jia S, Chen H, Luo S, et al. One-step approach to prepare superhydrophobic wood with enhanced mechanical and chemical durability: Driving of alkali. Applied Surface Science, 2018, 455: 115-122.
3. Xu L, Zhang H, Zheng C, et al. Enhancing the durability of reversible wettability on larch wood surfaces through optimized pretreatment methods. Industrial Crops and Products, 2024, 209: 118045.
4. Zhan K, Lu Q, Xia S, et al. A cost effective strategy to fabricate STA@PF@Cu_2O hierarchical structure on wood surface: Aimed at superhydrophobic modification. Wood Science and Technology, 2021, 55: 565-583.
5. Cao S, Cheng S, Wang P, et al. Construction and characterization of superhydrophobic wood

coatings using one-step technique. Colloid and Interface Science Communications, 2023, 57: 100757.

6. Lu Q, Cheng R, Jiang H, et al. Superhydrophobic wood fabricated by epoxy/Cu_2 $(OH)_3Cl$ NPs/stearic acid with performance of desirable self-cleaning, anti-mold, dimensional stability, mechanical and chemical durability. Colloids and Surfaces A: Physicochemical and Engineering Aspects, 2022, 647: 129162.
7. Tang W, Jian Y, Shao M, et al. A novel two-step strategy to construct multifunctional superhydrophobic wood by liquid-vapor phase deposition of methyltrimethoxysilane for improving moisture resistance, anti-corrosion and mechanical strength. Colloids and Surfaces A: Physicochemical and Engineering Aspects, 2023, 666: 131314.
8. Wang K, Dong Y, Yan Y, et al. Preparation of mechanical abrasion and corrosion resistant bulk highly hydrophobic material based on 3-D wood template. RSC advances, 2016, 6(100): 98248-98256.
9. Tan Y, Wang K, Dong Y, et al. Bulk superhydrophobility of wood via in-situ deposition of ZnO rods in wood structure. Surface and Coatings Technology, 2020, 383: 125240.

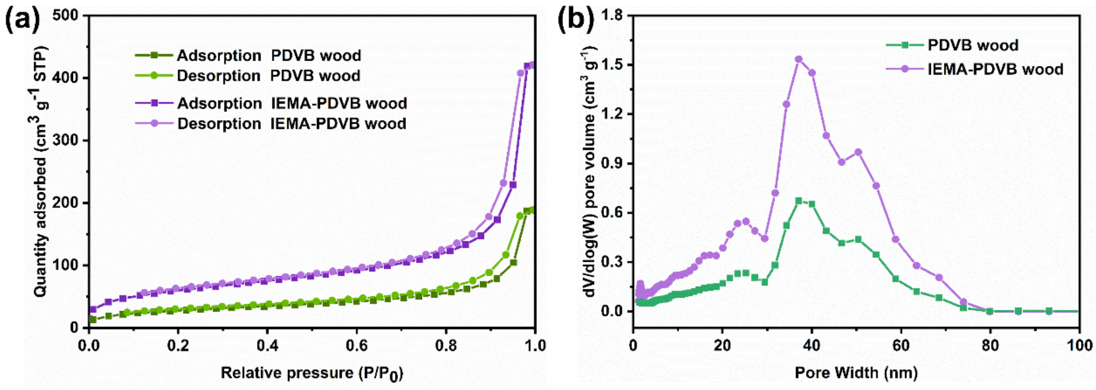

Fig. S1. (a) N_2 adsorption and desorption isotherms of PDVB wood and IEMA-PDVB wood; (b) Pore width distributions of PDVB wood and IEMA-PDVB wood.
